# Denitrification characterization of dissolved oxygen microprofiles in lake surface sediment through analyzing abundance, expression, community composition and enzymatic activities of denitrifier functional genes

**DOI:** 10.1186/s13568-019-0855-9

**Published:** 2019-08-19

**Authors:** Pei Hong, Xingqiang Wu, Yilin Shu, ChunBo Wang, Cuicui Tian, Shihao Gong, Pei Cai, Oscar Omondi Donde, Bangding Xiao

**Affiliations:** 1 Key Laboratory of Algal Biology of the Chinese Academy of Sciences, Institute of Hydrobiology, Chinese Academy of Sciences, No. 7 Donghu South Road, Wuhan, 430072 China; 2 University of Chinese Academy of Sciences, Beijing, 100049 China; 3 Key Laboratory for the Conservation and Utilization of Important Biological Resources of Anhui Province, Wuhu, 241000 China; 4 College of Life Sciences, Anhui Normal University, Wuhu, 241000 China

**Keywords:** Response surface methodology (RSM), DO concentration, Denitrifier, Lake surface sediment, Denitrification traits

## Abstract

The responses of denitrifiers and denitrification ability to dissolved oxygen (DO) concent in different layers of surface lake sediments are still poorly understood. Here, the optimal denitrification condition was constructed based on response surface methodology (RSM) to analyze the denitrification characteristics of surface sediments. The aerobic zone (AEZ), hypoxic zone (HYZ), up-anoxic zone (ANZ-1) and sub-anoxic zone (ANZ-2) were partitioned based on the oxygen contents, and sediments were collected using a customized-designed sub-millimeter scale sampling device. Integrated real-time quantitative PCR, Illumina Miseq-based sequencing and denitrifying enzyme activities analysis revealed that denitrification characteristics varied among different DO layers. Among the four layers, the DNA abundance and RNA expression levels of *norB*, *nirS* and *nosZ* were the highest at the aerobic layer, hypoxic layer and up-axoic layer, respectively. The hypoxia and up-anaerobic layer were the active nitrogen removal layers, since these two layers displayed the highest DNA abundance, RNA expression level and enzyme activities of denitrification functional genes. The abundance of major denitrifying bacteria showed significant differences among layers, with *Azoarcus*, *Pseudogulbenkiania* and *Rhizobium* identified as the main *nirS*, *nirK* and *nosZ*-based denitrifiers. Pearson’s correlation revealed that the response of denitrifiers to environmental factors differed greatly among DO layers. Furthermore, *napA* showed higher DNA abundance and RNA expression level in the aerobic and hypoxic layers than anaerobic layers, indicating that aerobic denitrifiers might play important roles at these layers.

**Electronic supplementary material:**

The online version of this article (10.1186/s13568-019-0855-9) contains supplementary material, which is available to authorized users.

## Introduction

Increased nitrogen (N, often in the form of nitrate) loading into aquatic environments has negative ecological and economic consequences on biodiversity and water quality (Dodds et al. 2009; Cardinale 2011). Denitrification processes in aquatic ecosystems act as a nitrate sink, transforming nitrate into gaseous products (N_2_, NO, N_2_O), which are then emitted into the atmosphere (Korom 1992; Verhoeven et al. 2006). Various metabolic enzymes, including nitrate reductases (Nar), nitrite reductases (Nir), nitric oxide reductases (Nor), and nitrous oxide reductases (Nos), catalyze the denitrification process (Zumft 1997). The denitrification functional genes *narG, napA*, *nirS*, *nirK*, *norB* and *nosZ* have commonly been used as biomarkers to elucidate the abundance, richness, and diversity of denitrifier communities (Tatti et al. 2015; Zhang et al. 2015; Yang et al. 2018).

Conventional biological denitrification requires hypoxic conditions with dissolved oxygen (DO) concentration less than 0.2 mg/L (Seitzinger et al. 2006). Since it was first discovered in the 1980s (Robertson and Kuenen 1984), aerobic denitrification at DO levels of 5.0–6.0 mg/L has attracted much attention because of its potential to overcome the disadvantages of conventional biological denitrification (Bai et al. 2019; Guo et al. 2016; Kim et al. 2008). Fluctuating oxygen concentrations, supply of nitrate, organic matter and other properties endow surface sediments (a few millimeters) a preferential place for denitrification (Santschi et al. 1990; Seitzinger et al. 2006). The denitrification characteristics in different habitats are always different, however, there is usually only one analysis method applied to investigate these (Yu et al. 2014; Saarenheimo et al. 2015; Tatti et al. 2015; Mao et al. 2017). Nevertheless, few studies have provided an integrated analysis of gene abundance, gene expression, enzyme activity and denitrifier community structure on a vertical scale within the micro-layers of lake sediment surfaces.

The global sedimentary denitrification rate has been found to be much lower (approximately 200 Tg a^−1^) than that of many existing measurement-based estimates (Devol 2015). This discrepancy may be as a result of scarcity of comprehensive measurements approaches. Hence, a comprehensive characterization of the denitrification process in lake surface sediments is needed to accurately evaluate the rate of denitrification yields and denitrification traits. Investigations are also important to understand the effects of DO content, temperature, pH and carbon source on denitrification (Strong et al. 2011; Kraft et al. 2014). Previous research have not reach a consensus in relation to that the effects of DO contents on different types of denitrification (Körner and Zumft 1989; Dalsgaard et al. 2014). Apart from DO, sediment physicochemical factors are also considered as important factors regulating lake denitrification (Saunders and Kalff 2001; Bruesewitz et al. 2011). However, there is little information regarding the interaction between denitrification characteristics and environmental factors among different DO sublayers.

Up to date, most studies utilized single-factor experiments; however, simultaneous changes in multiple environmental factors may impact nitrogen removal efficiency (Su et al. 2015). The conventional approach of assessing one factor at a time is not appropriate for this particular bioprocess because of potential interactions between independent variables. To overcome this problem, integration of multiple variables coupled with response surface methodology (RSM) should be used (Su et al. 2015). In the present study, surface sediments of a eutrophic lake and simulated artificial lake water were used to construct microcosms incubations. The optimal denitrification condition was constructed by adjusting the temperature, pH and organic carbon content (i.e., sawdust). Under the optimal conditions, different DO layers were sampled via a customised-designed sub-millimeter device to compare DNA abundance, RNA expression level and enzyme activity of denitrification enzymes. Moreover, the relationship between the sediment chemical factors and the denitrification processes within the vertical microecology was investigated. These results will help optimize conditions for nitrate removal from eutrophic water, and provide references for accurate assessment of denitrification ability of surface sediments.

## Materials and methods

### Preparation of sediments

Surface sediments were collected in October 2018 from Lake Dianchi, a eutrophic lake located in Kunming, P. R. China (24°40′–25°02′N, 102°36′–103°40′E), using the method described by Tian et al. (2015). Surface sediments were sealed in sterile plastic bags, transported to the laboratory, homogenized and then used for experiments.

### Experimental design for determination of nitrate removal rate under various conditions

Three temperatures (5 °C, 15 °C and 25 °C), three pH values (5.5, 7.0 and 8.5) and three sawdust contents (0.1, 0.3 and 0.5 mg/110 g of sediment) were set in the present study. Response Surface Methodology (RSM) combined with the Box-Behnken Design (BBD) were applied to test the effects of these three factors on nitrate removal rate. In total, 17 rounds of assays were conducted. Detailed settings of environmental conditions for each round of tests are listed in Additional file 1: Tables S1, S2. For assays, PVC cylinders (30 mm in diameter × 110 mm in height) were used to mimic aquatic ecosystems. In each cylinder, 110 g of sediments were placed at the bottom and then 30 mL of artificial lake water [48.6 mg/L NaNO_3_, 5.1 mg/L MgSO_4_·7H_2_O, 3.8 mg/L NH_4_Cl, 5.6 mg/L K_2_HPO_4_, 4.4 mg/L KH_2_PO_4_ and 0.1 mL/L trace elements (Nancharaiah et al. 2008)] was gently added above sediments. The apparatus was incubated at corresponding temperature under dark in an incubator (Hengfeng Medical Devices Co., Ltd. China). For each condition, 21 PVC cylinders were prepared. Five millilitre of water was sampled to determine nitrite content from three cylinders each day as three replicates. Content of nitrite in overlying water was immediately analyzed by ICS5000 chromelenon7 (Thermofisher, USA).

The experiments were continued until the nitrite content in water was below 1 mg/L. All experiments were finished within 7 days. The denitrification efficiency was calculated as the daily decrease of nitrite content from the initial value to the final value (the nitrite content observed below 1 mg/L for the first time).

### Sample preparation for determination of microbe indices under the optimal condition

**Fig. 1:**
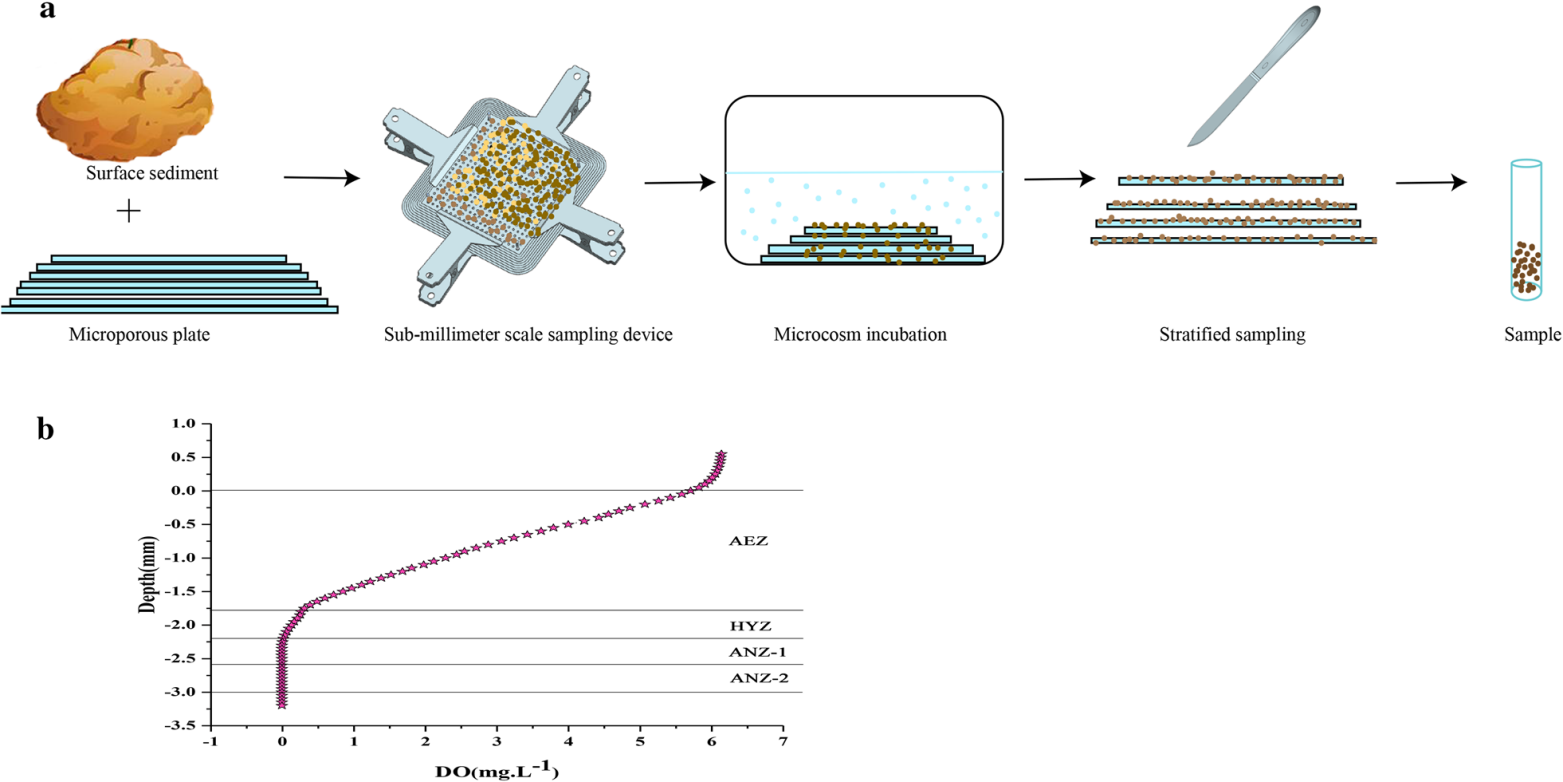
**a** Design of the sampling device used in the present study. **b** Dissolved oxygen profiles at different layers of sediments. *AEZ* aerobic zone, *HYZ* hypoxic zone, *ANZ-1* up-anoxic zone, *ANZ-2* sub-anoxic zone

To investigate expression levels of denitrification-related genes in different layers of sediments under the optimal environmental conditions, a special sub-millimeter sampling device was designed in the present study to accurately collect sediment samples at different depths (Fig. 1a). A series of different-sized microporous plates (0.2 mm thick, containing 256 pores with 3 mm diameter) were filled with sediments and then piled up. The size of upper plate was smaller than the lower one, forming a trapezoid structure. The upmost and nethermost plate was 8 cm × 8 cm and 11 cm × 11 cm in size, respectively. Overall, 20 microporous plates were stacked at the bottom of glass tanks (32 cm length × 20 cm width × 10 cm height), and then immersed in artificial lake water (total water depth was 8 cm). These tanks were incubated under dark at 25 °C in incubators. After stabilized for 2 days, changes of DO content in sediments along with depth were determined using an oxygen microsensor (Fig. 1b). Based on the DO contents, four layers of sediments were defined, including aerobic zone (AEZ, 0–1.8 mm depth, DO: 0.2–5.9 mg L^−1^), hypoxic zone, HAZ (1.8–2.2 mm depth, DO: 0–0.2 mg L^−1^); up-anoxic zone (ANZ-1, 2.2–2.6 mm depth, DO: 0 mg L^−1^) and sub-anoxic zone (ANZ-2, 2.6–3.0 mm depth, DO: 0 mg L^−1^). After incubation for 5 days, sediments were collected from these zones and stored at − 80 °C for biochemical and molecular analyses.

### Analyses of chemical parameters in sediments

NH_4_^+^–N, NO_3_^−^–N and NO_2_^−^–N were extracted from sediments using 2 mol/L KCL solution at a ratio of 1: 5 (sediment: water) and measured using ICS5000 chromelenon7 (Thermofisher, USA). Frozen dried sediments were sieved and then analyzed for total organic carbon contents (TOC) using an Elementar vario TOC system (Elementar, Germany) and TN was analyzed by hydrochloric acid photometry method. All parameters were measured in triplicates.

### Determination of activities of denitrification enzymes and electron transport system (ETS)

Methods for detecting denitrification enzyme activities and electron transport system (ETS) activity followed Su et al. (2019). Briefly, 5 g of sediments were suspended in 100 mM phosphate-buffered saline (PBS, pH 7.8) and then sonicated at 4 °C for 5 min to break cells. After centrifugation at 16,000 rpm for 10 min at 4 °C, the supernatants were collected for determination of NAR, NIR and NOS activities. The assay mixture (3 mL) included 10 mM PBS buffer (pH 7.8), 5 mM Na_2_S_2_O_4_, 10 mM methyl viologen, 1 mM denitrifying electron acceptor (NO_3_^−^, NO_2_^−^ or N_2_O) and 1 mL of enzyme extract. After incubation at 25 °C under anaerobic conditions for 30 min, the increased or decreased NO_2_^−^ concentration was determined at 540 nm to calculate NAR and NIR activities. The reduced N_2_O concentration was detected by a microsensor (MMM-Meter, Unisense, Denmark) to calculate NOS activities. Reduction from 2-(p-iodophenyl)-3-(p-nitrophenyl)-5- phenyl tetrazolium chloride (INTC) to formazan caused by enzyme extract was determined to express ETS activity.

### Nucleic acid extraction and real-time quantitative PCR (RT-qPCR)

DNA was extracted from approximately 0.8 g of each sediment sample using an E.Z.N.A. Soil DNA Kit (Omega Bio-Tek, Norcross, GA, USA) according to the manufacturer’s instructions. Total RNA was extracted using the acid guanidium thiocyanate-phenol–chloroform (AGPC) method (Choi et al. 2018). After extraction, RNA was reversely transcribed into cDNA using Superscript II reverse transcriptase (Life Technologies Corporation, USA) in accordance with manufacturer’s instructions. Nucleic acid quality and concentration were examined by 1% agarose gel electrophoresis and spectrophotometry, respectively.

DNA levels and RNA transcriptional levels of 16 s rRNA, *narG*, *norB*, *nirS*, *nirK*, *nosZ* and *napA* were examined in the extracted DNA and RNA using the RT-qPCR method and then expressed as copies per gram of sediment.

The primers and conditions for RT-qPCR are provided in Additional file 1: Table S3. RT-qPCR experiments were performed on Bio-Rad qPCR machine (Hercules, CA, USA) using SYBR Green as the signal dye. Each 20-μL reaction mixture contained 1 μL of template DNA, 10 μL of iTaq Universal SYBR Green Supermix (Bio-Rad), 1 μL of 10 µM each primer, and 7 μL of water. Standard curves for each gene were obtained by tenfold serial dilution of standard plasmids containing target functional gene. Positive (plasmid DNA only) and negative (nuclease-free water) controls were prepared simultaneously. The ratio of DNA level to RNA transcriptional level for each gene was calculated and expressed as ratio of RNA to DNA (RNA: DNA).

### High-throughput sequencing

PCR products of *nirS*, *nirK* and *nosZ* were amplified from DNA samples. The primers and conditions are presented in Additional file 1: Table S3. Amplicons were purified, pooled in equimolar concentrations for paired-end sequencing (2 × 300 bp) on an Illumina MiSeq platform (Illumina, San Diego, CA, USA) by LC-Bio Technology Company (Hangzhou, China) according to standard protocols. Operational taxonomic units (OTUs) were clustered with 97% similarity cutoff using UCHIME (version 7.1, http://drive5.com/uparse/), which also identified and removed chimeric sequences. Representative sequences were selected for each OTU, and taxonomy information of each representative sequence was obtained using the RDP Classifier (http://rdp.cme.msu.edu/) by blasting against the functional gene database (FGR, Fish et al. 2013). Beta diversity was calculated by analysis of similarities (ANOSIM) with weighted Unifrac in the R “vegan” package (v3.2.3).

### Statistical analysis

Analysis of Variance (ANOVA) and Pearson’s correlation analysis were conducted using SPSS 16.0 software. Statistical significance was set at the *P* level of < 0.05. Figures were drawn using the Origin 8.0 program.

## Results and discussion

### Optimal environmental conditions for denitrification of water

**Fig. 2:**
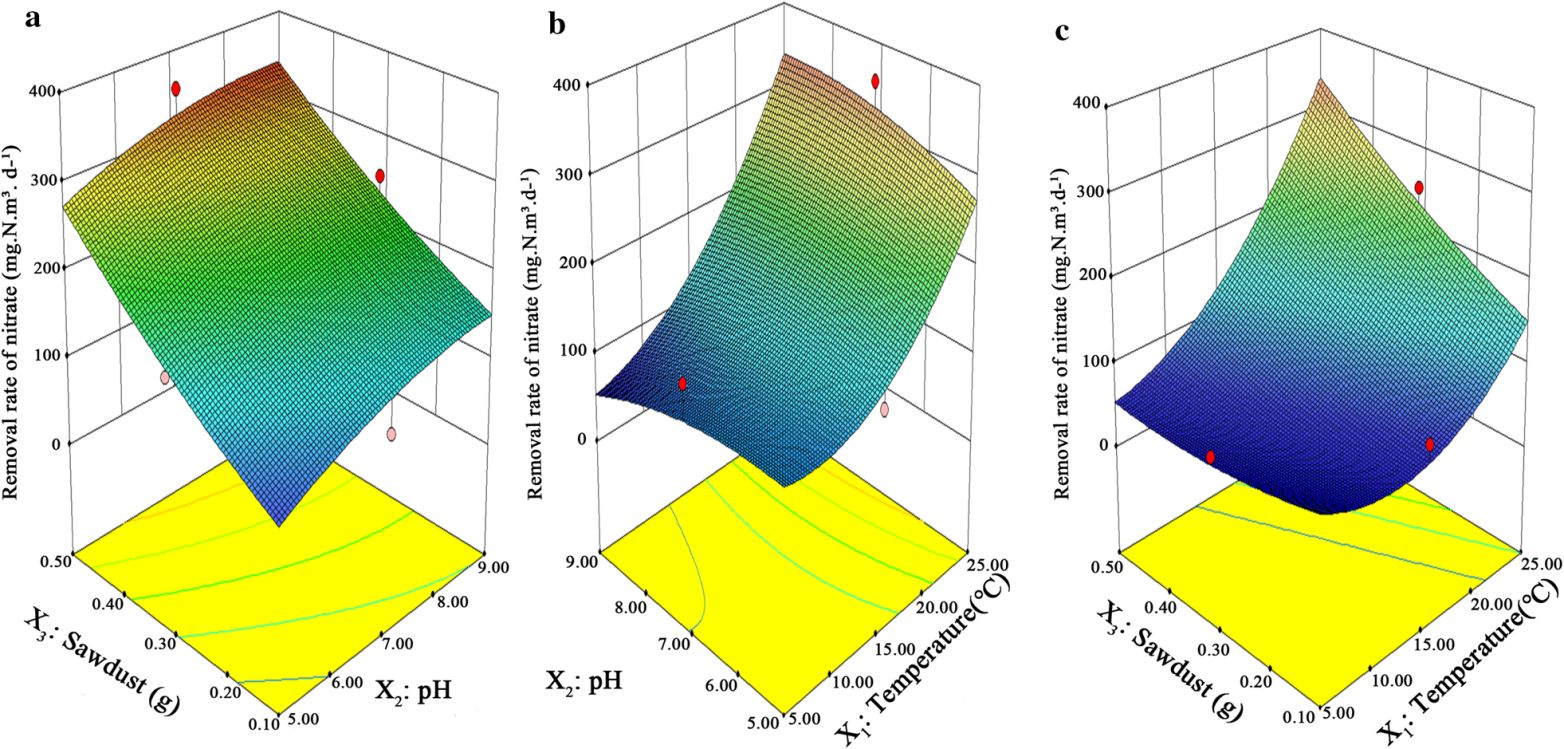
Removal efficiency of nitrate under different conditions. **a** Changes of nitrate removal efficiency in response to treatments with different sawdust contents and pH; **b** changes of nitrate removal efficiency in response to treatments with different temperatures and pH; **c** changes of nitrate removal efficiency in response to treatments with different sawdust contents and temperature

Previous studies have investigated the effects of environmental factors on sediment denitrification (Huang et al. 2017; Myrstener et al. 2016; Saarenheimo et al. 2015). However, few studies have applied RSM to evaluate the interactive effects of environmental factors (temperature, pH and availability of organic C) on nitrate removal. In the present study, the interaction of temperature, sawdust content and pH on the removal of nitrate nitrogen caused by surface sediments were analyzed based on RSM. Additional file 1: Table S2 presents the determined nitrate removal rate under various conditions and Fig. 2 shows the response surface of the nitrate removal efficiency at different sawdust contents, pH values and temperatures. These results suggested that sawdust content, pH and temperature all significantly and positively affected nitrate removal rate in water (Fig. 2a, c).

ANOVA for response surface quadratic model revealed that F-value of the model was equal to 4.03 and the *P* value of the lack of fit was higher than 0.05 (Additional file 1: Table S4), suggesting that the as-obtained model was statically significant (Additional file 1: Table S4), which could be used to predict the optimal denitrification conditions. Besides, ANOVA revealed that temperate, sawdust content and their interaction all significantly affected nitrate removal efficiency (P = 0.0083, Additional file 1: Table S4). High temperature should accelerate growth of microorganisms and increase denitrification activities, thus increasing nitrate removal efficiency. In the present study, addition of sawdust promoted nitrate removal efficiency, which was consistent with previous findings (Wang and Chu 2016). The contribution of the three tested variables to denitrification efficiency followed the order temperature > sawdust content > pH, and the optimum condition for maximum nitrate removal were predicted as temperature = 25 °C, pH = 8.5 and sawdust content = 0.5 mg/110 g of sediment.

### Abundance, transcriptional levels, enzyme activities of denitrifiers in surface sediments

**Fig. 3:**
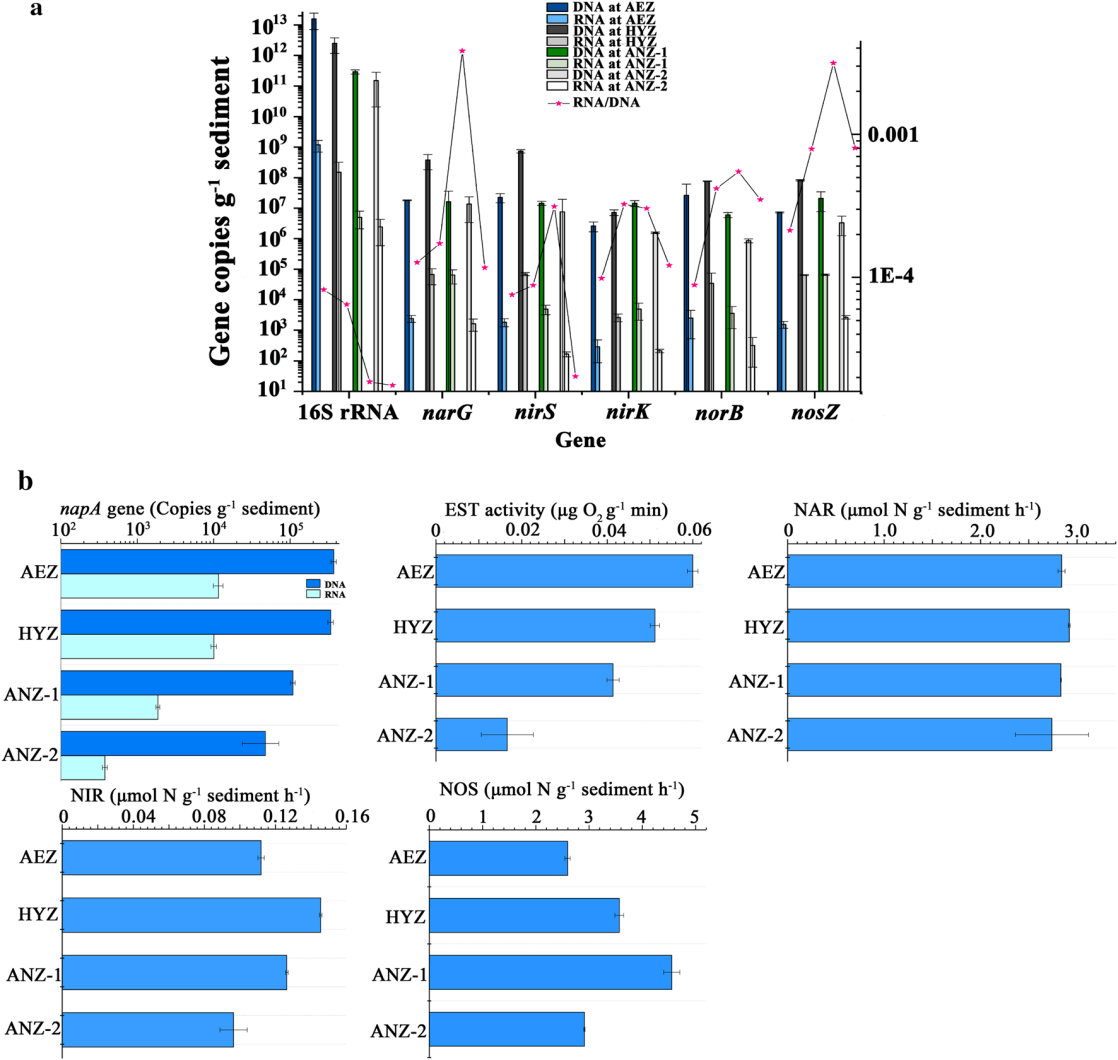
**a** Copies of 16S rRNA, *narG*, *nirK*, *nirS*, *norB* and *nosZ* genes per gram of sediment at DNA level, RNA level and their ratios (ratio of copies at RNA level to that at DNA level for each gene, RNA: DNA). **b** Copies of *napA* gene per gram of sediment, activities of electron transport system (ETS) and denitrifying enzymes at different layers of sediments. Error bars indicate standard errors of three replicates. *NAR* nitrate reductase, *NIR* nitrite reductase, *NOS* nitric oxide reductase, *AEZ* aerobic zone, *HYZ* hypoxic zone, *ANZ-1* up-anoxic zone, *ANZ-2* sub-anoxic zone

Under the optimal nitrate removal condition, denitrifier abundance (at DNA level), transcriptional levels (at RNA level) and enzyme activities were compared among different layers of surface sediments (AEZ, HAZ, ANZ-1 and ANZ-2). All the tested genes were detected in all samples at both DNA and RNA levels, suggesting that the whole denitrification process took place in all these layers of sediments. However, the abundance and transcriptional levels of different denitrifying genes differed among layers. The order of the denitrifying gene abundance in AEZ, HAZ, ANZ-1 and ANZ-2 was *norB *> *nirS *> *narG *> *nosZ *> *nirK*, *nirS *> *narG *> *nosZ *> *norB *> *nirK*, *nosZ *> *narG *> *nirS *> *nirK *> *norB*, *narG *> *nirS *> *nosZ *> *nirK *> *norB*, respectively (Fig. 3a).

In addition, since *napA* is an indicator gene of aerobic denitrification (Marchant et al. 2017), we also compared its distribution among the four sediment layers. In this study, the DNA abundance and RNA transcriptional level of *napA* in the aerobic and hypoxic regions were significantly higher than those in the anoxic regions (Fig. 3b). Therefore, accurate understanding of the range of aerobic denitrification layer and activities of aerobic denitrification bacteria.

Denitrification was restricted to a narrow zone immediately below the aerobic–anaerobic interface in sediments and biofilms (Deutzmann et al. 2014). Previous studies have investigated areas of denitrification using microsensors to detect oxygen and nitrate concentrations in sediments. However, little information is available regarding determination of the dominant denitrification region in lake surface sediments based on abundance, expression, community composition and enzymatic activities of denitrifier functional genes (Christensen et al. 1989; Nielsen et al. 1990a, b). In the present study, among the four sediment layers, RNA transcriptional levels of *narG*, *nirS*, *nirK*, *norB* and *nosZ* were relatively higher in HYZ and ANZ-1 than those in AEZ and ANZ-2 (Fig. 3a). Besides, enzyme activities of NIR and NOS also showed similar trends. These results indicated that the hypoxic layer and the up-anaerobic layer were the active nitrogen removal layers.

Comparison of the DNA abundance and RNA transcriptional level of denitrification genes between the up- (HYZ-1) and sub-anoxic layers (HYZ-2) revealed a decreasing trend with depth, which might be due to the decreased total bacteria in the sub-anoxic layers. Lower copy number of 16S rRNA and EST activity were observed in HYZ-2 (Fig. 3b). This phenomenon was consistent with the decreases of total bacteria abundance with depth in other sediments (Qin et al. 2018). These findings further demonstrated that not all anoxic vertical profiles were active denitrification regions.

### Vertical distribution for representative denitrifier communities in surface sediments

**Fig. 4:**
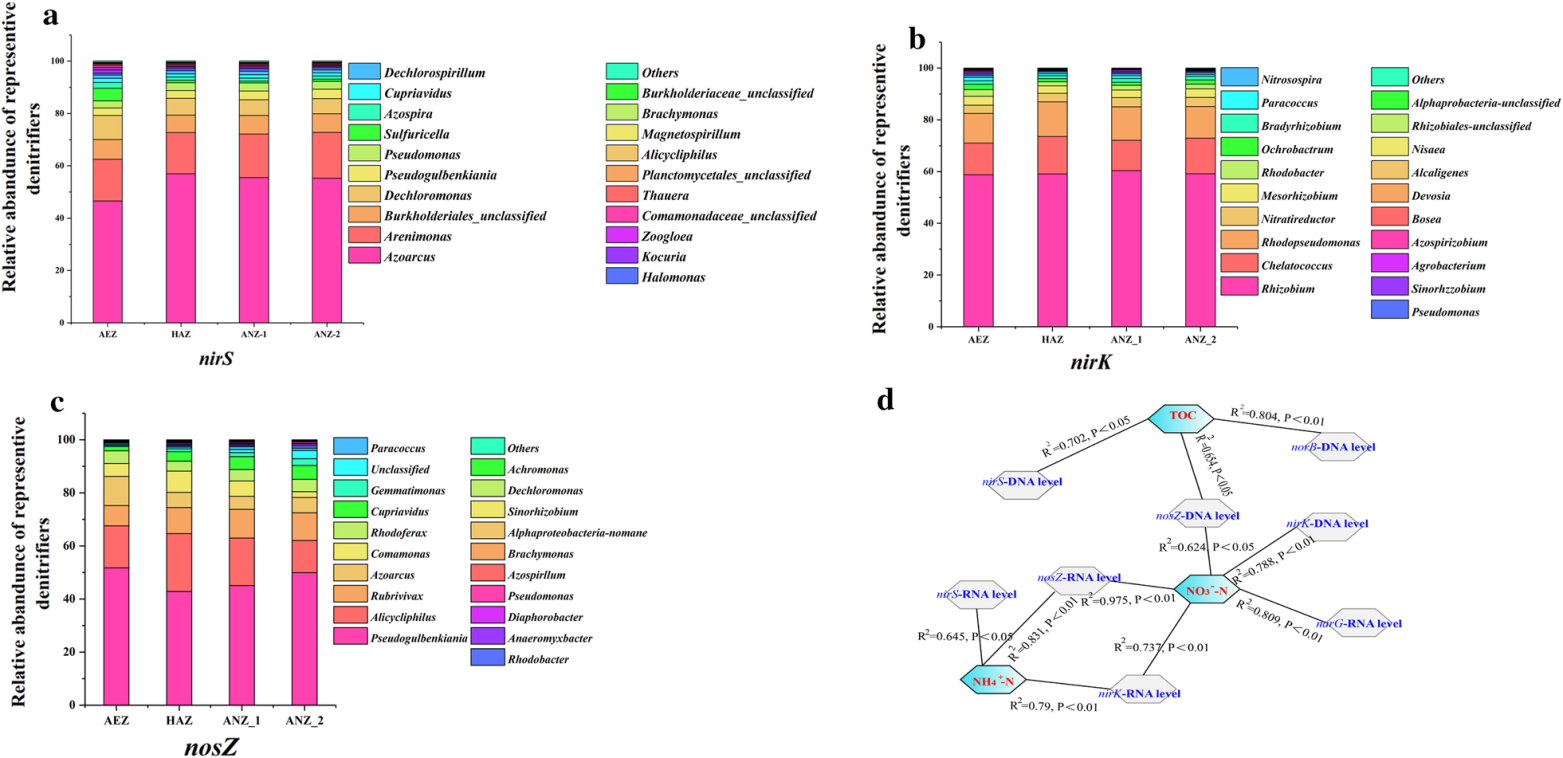
Community structures in different layers of sediments based on sequence analysis of *nirS* (**a**), *nirk* (**b**) and *nosZ* (**c**). **d** Pearson correlation matrix between DNA abundance, RNA transcriptional level of denitrification genes and sediment physicochemical factors. *AEZ* aerobic zone, *HYZ* hypoxic zone, *ANZ-1* up-anoxic zone, *ANZ-2* sub-anoxic zone

Blasting of *nirS*, *nirK* and *nosZ* sequences to FGR databases enabled taxonomic analyses of the denitrifier communities among different sediment layers, which has also been applied in other studies (such as Yang et al. 2018). ANOSIM revealed significant differences in denitrifier community structure among vertical profiles (*nirS*, *R*^2^ = 0.35, *P *= 0.02; *nirK*, *R*^2^ = 0.37, *P *= 0.04; *nosZ*, *R*^2^ = 0.67, *P *= 0). In total, 11,171, 7007 and 3063 OTUs were identified for *nirS*-type, *nirK*-type and *nosZ*-type denitrifiers, respectively. Dominant *nirS*, *nirK* and *nosZ* OTUs were identified to be the genera *Azoarcus*, *Rhizobium* and *Pseudogulbenkiania* in the four sediment layers (Fig. 4a–c), respectively. Each layer showed significant differences in the types and abundances of denitrifiers (Additional file 1: Table S5). Furthermore, in the aerobic layer, comparison of the abundance of the top five genus among *nirS*-type, *nirK*-type and *nosZ*-type denitrifiers revealed higher abundance of *Dechloromonas* and *Azoarcus* in AEZ than those in other three layers, and higher abundance of *Pseudogulbenkiania* than that in hypoxic layer. Previous studies reported a high abundance of *Dechloromonas* in agricultural soils and reservoirs (Coyotzi et al. 2017; Yu et al. 2014), as well as high levels of *Azoarcus* in oilfields, wastewater treatment plants, soils and sediments (Song et al. 2000; Wang et al. 2014a; Nazina et al. 2017), and high abundance of *Pseudogulbenkiania* in freshwater sediments and rice paddy soils (Tago et al. 2011; Guo et al. 2018). In addition, most of the isolated denitrifier strains in these genera were anaerobic strains. However, there was no much information pertaining to the isolation of aerobic denitrifier strains (Achenbach et al. 2001; Ishii et al. 2016; Yücel et al. 2019). These results indicated that a lot of aerobic denitrifier strains have not been isolated from the aerobic layer, which might be used for in situ restoration of eutrophic lake.

The abundance of *Azoarcus* was higher than other genera in the hypoxic layer. Previous studies showed that bacteria in the genus *Azoarcus* could use many aromatic hydrocarbons as carbon sources during denitrification processes (Zhou et al. 1995; Springer et al. 1998; Lee et al. 2013). Therefore, the genus *Azoarcus* might be the dominant denitrifying bacteria for nitrogen removal in low-DO sediment areas. Analysis of *nosZ* gene sequences showed that the abundance of *Pseudogulbenkiania* was higher than those of other bacteria in the anoxic layer. Previous studies presented some isolated strains of *Pseudogulbenkiania* and showed strong denitrification and N_2_O reduction activities in rice paddy soils (Tago et al. 2011; Yoshida et al. 2012). Therefore, *Pseudogulbenkiania* might be the most important N_2_O reducing microbes in the anoxic layer of surface sediments.

### Relationship between denitrification traits and sediment physicochemical factors

To date, several studies have investigated the spatial changes of denitrifier traits in sediments (Devol 2015; Mao et al. 2017; Zhang et al. 2015). However, little is known about the relationship between denitrifier traits and physicochemical factors in surface sediments. In the present study, one-way ANOVA showed that the TN content (P < 0.01), TOC content (P < 0.01), NH_4_^+^–N content (P < 0.01), and NO_3_^−^–N content (P < 0.01) differed significantly among AEZ, HAZ, ANZ-1 and ANZ-2 (Additional file 1: Table S6). Pearson’s correlation revealed that the NH_4_^+^–N, NO_3_^−^–N and TOC content was significantly positively correlated with DNA abundance and RNA transcriptional level of denitrification genes (Fig. 4d). These results indicated that the response of denitrifiers to physicochemical factors varied in different DO layers. Similarly, Wang et al. (2014b) also revealed that physicochemical factors markedly affected the distribution of denitrification bacteria in bay sediments (Wang et al. 2014b). Besides, different genes revealed inconsistent relationship between physicochemical factors and the abundance of denitrification genes. Similar inconsistence was also reported in marine sediments (Gao et al. 2017).

In summary, following the RSM experiments, the optimal environmental conditions for best nitrate removal in water was predicted as 25C, pH 8.5 with 0.5 mg sawdust/110 g of sediment. Under the optimal environmental conditions, DNA abundance, RNA transcriptional levels and enzyme activities were compared among different layers of surface sediments, revealing that the activities of denitrification enzymes and key denitrifiers varied among layers with different DO contents. The as-obtained relationship between denitrification and environmental factors improved the understanding of their roles in geobiochemical cycles of Nitrogen.

## Additional file

**Additional file 1.** Additional tables.

## Data Availability

The raw Illumina reads obtained in the current study were deposited in the NCBI short-read archive under SRA Accession PRJNA525978 (https://www.ncbi.nlm.nih.gov/sra/PRJNA525978).
