# Supplementary material for: Denitrification characterization of dissolved oxygen microprofiles in lake surface sediment through analyzing abundance, expression, community composition and enzymatic activities of denitrifier functional genes
Source: AMB Express. 2019 Aug 19;9:129. doi: 10.1186/s13568-019-0855-9 (PMC6702497; doi:10.1186/s13568-019-0855-9)
Supplement: Supplementary file 1 — Additional file 1. Additional tables. [file 13568_2019_855_MOESM1_ESM.docx]

**AMB Express**

**A novel revelation on dissolved oxygen microprofile denitrification characterization in the lake sediment surface through denitrifier functional gene abundance, expression, community composition and enzymatic activities analysis**

Pei Hong ^1,2^, Xingqiang Wu^1,2^, Yilin Shu^3,4^, ChunBo Wang ^1,2^,Cuicui Tian^1,2^, Shihao Gong^1,2^, Pei Cai^1,2^ Oscar Omondi Donde^1,2^ and BangdingXiao^1,2^*

1. Key Laboratory of Algal Biology of the Chinese Academy of Sciences, Institute of Hydrobiology, Chinese Academy of Sciences, Wuhan 430072, China.

2. University of the Chinese Academy of Sciences, Beijing 100049, China.

3. Key Laboratory for the Conservation and Utilization of Important Biological Resources of Anhui Province, Wuhu 241000, China.

4. College of Life Sciences, Anhui Normal University, Wuhu 241000, China.

* Correspondence : Prof. Bangding Xiao, Institute of Hydrobiology, Chinese Academy of Sciences, No.7 Donghu south road, Wuhan 430072, China. Tel: +86 27 68780386; Fax: +86 27 68780712, [bdxiao618@163.com](mailto:bdxiao618@163.com)

Authors email addresses:

Pei Hong, [sjhongpei@163.com](mailto:sjhongpei@163.com);

Xingqiang Wu, xqwu@ihb.ac.cn

Yilin shu, [sjshuyilin@163.com](mailto:sjshuyilin@163.com);

ChunBo Wang, wangchunbo@ihb.ac.cn

Cuicui Tian, tiantian5629@126.com

Shihao Gong, [gongshihao941214@outlook.com](mailto:gongshihao941214@outlook.com)

Pei Cai, [caipei@ihb.ac.cn](mailto:caipei@ihb.ac.cn)

Oscar Omondi Donde, oscinho@yahoo.co.uk

BangdingXiao, [bdxiao618@163.com](mailto:bdxiao618@163.com)

Additional file 1: Table S1 Experimental ranges and levels of the independent variables for response surface methodology (RSM) model.

| **Variables** | **Symbols** | **Real values of coded levels**^a^ | | |
| --- | --- | --- | --- | --- |
|  |  | -1 | 0 | 1 |
| Temperature (℃) | X_1_ | 5 | 15 | 25 |
| Initial pH | X_2_ | 5.5 | 7 | 8.5 |
| Sawdust content (mg/110g sediment) | X_3_ | 0.1 | 0.3 | 0.5 |

^a^The center point was replicated five times to estimate the experimental errors.

Additional file 2: Table S2 Box-Behnken design matrix, corresponding experimental and predicted responses of nitrate removal efficiency as affected by temperature, pH, sawdust content.

| Run | Temperature  (variable: X_1_) | pH (variable: X_2_) | Sawdust   (variable: X_3_) | Nitrate removal rate (mg.N.m^-3^.d^-1^)  (Response) |
| --- | --- | --- | --- | --- |
| 1^a^ | 15(0) | 8.5(1) | 0.1(-1) | 79.48 |
| 2 | 15(0) | 7(0) | 0.3(0) | 73.4 |
| 3 | 15(0) | 5(-1) | 0.5(1) | 111.38 |
| 4 | 5(-1) | 5.5(-1) | 0.3(0) | 83.47 |
| 5 | 25(1) | 7(0) | 0.1(-1) | 88.23 |
| 6 | 15(0) | 5.5(-1) | 0.1(-1) | 85.94 |
| 7 | 5(-1) | 7(0) | 0.1(-1) | 89.68 |
| 8 | 25(1) | 5.5(-1) | 0.3(0) | 150.47 |
| 9 | 15(0) | 7(0) | 0.3(0) | 116 |
| 10 | 5(-1) | 7(0) | 0.5(1) | 139.46 |
| 11 | 15(0) | 7(0) | 0.3(0) | 120.93 |
| 12 | 5(-1) | 8.5(1) | 0.3(0) | 65.09 |
| 13 | 15(0) | 7(0) | 0.3(0) | 108.55 |
| 14 | 15(0) | 7(0) | 0.3(0) | 64.35 |
| 15 | 15(0) | 8.5(1) | 0.5(1) | 88.07 |
| 16 | 25(1) | 8.5(1) | 0.3(0) | 251.48 |
| 17 | 25(1) | 7(0) | 0.5(1) | 353.86 |

^a^The center point was replicated five times to estimate the experimental errors.

Additional file 3: Table S3 Primers and conditions for PCR analyses.

|  | **Target gene** | **Primer** | **5’-3’** | **PCR cycling profile** | **r^2^** | **Amplification efficiency (%)** | **Reference** |  |
| --- | --- | --- | --- | --- | --- | --- | --- | --- |
| qPCR and qRT-PCR | 16S rRNA | 341F | CCTACGGGAGGCAGCAG | 95°C/4min, 94°C/45s, 65°C/20s, 40cycles | 0.914 | 92.4 | Hegler et al. 2012 | Hegler, F., et al. "Influence of Seasonal and Geochemical Changes on the Geomicrobiology of an Iron Carbonate Mineral Water Spring." Applied and Environmental Microbiology 78.20(2012):7185-7196. |
|  |  | 797R | GGACTACCAGGGTATCTAATCCTGTT |  |  |  |  |  |
|  | narG | F | TCGCCSATYCCGGCSATGTC | 94°C/2min, 94°C/15s, 59°C/30s, 72°C /1min, 40cycles | 0.963 | 101.5 | Su et al. 2019 | Su et al., Impacts of chlorothalonil on denitrification and N2O emission in riparian sediments: microbial metabolism mechanism.2019 Water Res 148: 188-197. |
|  |  | R | GAGTTGTACCAGTCRGCSGAYTCSG |  |  |  |  |  |
|  | norB | 2F | GACAAGNNNTACTGGTGGT | 94°C/2min, 94°C/15s, 56°C/30s, 72°C /1min, 40cycles | 0.984 | 99.6 | Su et al. 2019 | Su et al., Impacts of chlorothalonil on denitrification and N2O emission in riparian sediments: microbial metabolism mechanism.2019 Water Res 148: 188-197. |
|  |  | 6R | GACAAGNNNTACTGGTGGT |  |  |  |  |  |
|  | nirS | cd3aF | AACGYSAAGGARACSGG | 94°C/2min, 94°C/15s, 57°C/30s, 72°C /1min,40cycles | 0.995 | 104.8 | Kandeler et al. 2006 | Kandeler, E., et al., Abundance of narG, nirS, nirK, and nosZ Genes of Denitrifying Bacteria during Primary Successions of a Glacier Foreland. Applied and Environmental Microbiology, 2006. 72(9): p. 5957-5962. |
|  |  | R3cd | GASTTCGGRTGSGTCTTSAYGAA |  |  |  |  |  |
|  | nirK | 876F | ATYGGCGGVCAYGGCGA | 95°C/5min, 95°C/15s, 66.4°C/30s, 72°C /1min, 40cycles | 0.957 | 112.6 | Henry et al. 2004 | Henry, S., et al., Quantification of denitrifying bacteria in soils by nirK gene targeted real-time PCR. Journal of Microbiological Methods, 2004. 59(3): p. 327-335. |
|  |  | 1040R | GCCTCGATCAGRTTRTGGTT |  |  |  |  |  |
|  | nosZ | 1F | WCSYTGTTCMTCGACAGCCAG | 95°C/5min, 95°C/15s, 64.4°C/1min, 72°C /1min, 40cycles | 0.958 | 90.1 | Henry et al. 2006 | Henry, S., et al., Quantitative Detection of the nosZ Gene, Encoding Nitrous Oxide Reductase, and Comparison of the Abundances of 16S rRNA, narG, nirK, and nosZ Genes in Soils. Applied and Environmental Microbiology, 2006. 72(8): p. 5181-5189. |
|  |  | 1R | ATGTCGATCARCTGVKCRTTYTC |  |  |  |  |  |
|  | napA | z3F | CGCGAACAAGCTGATGAAGG | 95°C/5min, 95°C/15s, 60°C/1min, 72°C /1min, 40cycles | 0.912 | 110.3 | Gui M et al., 2017 | Gui M et al., Effect of NaCl on aerobic denitrification by strainAchromobactersp. GAD-3[J]. Applied Microbiology and Biotechnology, 2017, 101(12):5139-5147. |
|  |  | z3R | AAGATCATCGGGATGTCGGC |  |  |  |  |  |
| Illumina MiSeq sequencing | nirS | cd3aF | AACGYSAAGGARACSGG | 98°C/ 30s, 98°C /10 seconds, 50 /30 s, 72°C/45 s, 35cycles, 72°C/10 min |  |  | Kandeler et al. 2006 | Kandeler, E., et al., Abundance of narG, nirS, nirK, and nosZ Genes of Denitrifying Bacteria during Primary Successions of a Glacier Foreland. Applied and Environmental Microbiology, 2006. 72(9): p. 5957-5962. |
|  |  | R3cd | GASTTCGGRTGSGTCTTSAYGAA |  |  |  |  |  |
|  | nirK | F1aCu | ATCATGGT(C\G)CTGCCGCG | 98°C/ 30s, 98°C /10 seconds, 53 /30 s, 72°C/45 s, 35cycles, 72°C/10 min |  |  | Throback et al. 2004 | Throback, IN., et al., Reassessing PCR primers targeting nirS, nirK and nosZ genes for community surveys of denitrifying bacteria with DGGE. FEMS Microbiol Lett (2004) 49: 401–417. |
|  |  | R3Cu | GCCTCGATCAG(A/G)TTGTGGTT |  |  |  |  |  |
|  | nosZ | F | GGGCTBGGGCCRTTGCA | 98°C/ 30s, 98°C /10 seconds, 58 /30 s, 72°C/45 s, 35cycles, 72°C/10 min |  |  | Chen et al. 2012 | Chen, Z., et al., Differentiated response of denitrifying communities to fertilization regime in paddy soil[J]. Microbial Ecology, 2011, 63(2):446-459. |
|  |  | R | GAAGCGRTCCTTSGARAACTTG |  |  |  |  |  |

Additional file 4: Table S4 Analysis of variance (ANOVA) for response surface quadratic model (Y)^a^.

| **Sources** | **Mean Square** | **F-value** | **P-value** | **Statistics** |
| --- | --- | --- | --- | --- |
| Model ^b^ | 8272.36 | 4.03 | 0.0398 | significant |
| X_1_ | 27183.07 | 13.24 | 0.0083 | significant |
| X_2_ | 349.27 | 1.70E-01 | 0.6923 | NS |
| X_3_ | 15264.56 | 7.44 | 0.0295 | significant |
| X_1_X_2_ | 3563.65 | 1.74 | 0.2291 | NS |
| X_1_X_3_ | 11646.78 | 5.67 | 0.0487 | significant |
| X_2_X_3_ | 70.95 | 0.035 | 0.8578 | NS |
| X_1_^2^ | 14549.98 | 7.09 | 0.0324 | significant |
| X_2_^2^ | 1334.85 | 0.65 | 0.4465 | NS |
| X_3_^2^ | 644.8 | 0.31 | 0.5926 | NS |
| Residual | 205.822 |  |  |  |
| Lack of fit | 3898.41 | 5.79 | 0.0614 | NS |
| Pure error | 6.27E+02 |  |  |  |
| Cor total | 8272.36 |  |  |  |

NS: not significant. ^a^ The results were generated from the Design Expert software (version 8.0).

^b^ X_1_ is Temperature (℃). X_2_ is pH. X_3_ is Sawdust content.

Additional file 5: Table S5 Significant difference distribution of the top five denitrifying bacteria among vertical layers.

|  | Denitrifier community | Abundance comparison among layers | *P*-value |
| --- | --- | --- | --- |
| *nirS*-type | *Azoarcus* | HYZ>AEN | 0.026 |
|  | *Dechloromonas* | AEN>HYZ>ANZ-1>ANZ-2 | 0.05 |
| *nirK*-type | *Rhodopseudomonas* | HYZ>AEN | 0.028 |
| *nosZ*-type | *Pseudogulbenkiania* | AEN>HYZ | 0.028 |
|  | *Alicycliphilus* | HYZ>AEN | 0.01 |
|  | *Rubrivivax* | ANZ-1>ANZ-2>AEN>HYZ | 0.022 |
|  | *Azoarcus* | AEN>ANZ-2>ANZ-1>HYZ | 0.001 |
|  | *Comamonas* | HYZ>AEN | 0.044 |
|  |  |  |  |

Significant differences among four layers as determined by one-way ANOVA method (LSD, p < 0.05).

Additional file 6: Table S6 Vertical distribution and one-way ANOVA of the main chemical properties in sediments.

| **Depth (mm)** | **TN(g/kg)** | **TOC(g/kg)** | **NH_4_^+^-N(mg/kg)** | **NO_3_^-^-N(mg/kg)** |
| --- | --- | --- | --- | --- |
| 0-1.8 (AEZ) | 3.596±0.0017(a) | 18.09±0.02940(a) | 32.36±0.00013(a) | 1.2407±0.03461(a) |
| 1.8-2.2 (HAZ) | 4.3176±0.00008(b) | 20.47±0.05288(b) | 34.103±0.00164(b) | 3.70635±0.00938(b) |
| 2.2-2.6 (ANZ-1) | 4.9904±0.00032(c) | 10.12±0.01268(c) | 35.77±0.00017(c) | 3.8334±0.01306(c) |
| 2.6-3.0 (ANZ-1) | 3.5031±0.00221(d) | 8.1106±0.071303(d) | 33.44±0.00739(d) | 2.14786±0.06839(d) |
|  |  |  |  |  |

TN and TOC represent total nitrogen and total organic carbo, respectively. Data are the means ± standard error (n = 3). Different lowercase letters (a, b, c, d) represent significant differences among four layers as determined by one-way ANOVA method (LSD, p < 0.05).
